# Genomewide Analysis of Inherited Variation Associated with Phosphorylation of PI3K/AKT/mTOR Signaling Proteins

**DOI:** 10.1371/journal.pone.0024873

**Published:** 2011-09-19

**Authors:** Janna E. Hutz, W. Aaron Manning, Michael A. Province, Howard L. McLeod

**Affiliations:** 1 Institute for Pharmacogenomics and Individualized Therapy, University of North Carolina at Chapel Hill, Chapel Hill, North Carolina, United States of America; 2 Division of Statistical Genomics, Washington University in St. Louis, St. Louis, Missouri, United States of America; Universite de Montreal, Canada

## Abstract

While there exists a wealth of information about genetic influences on gene expression, less is known about how inherited variation influences the expression and post-translational modifications of proteins, especially those involved in intracellular signaling. The PI3K/AKT/mTOR signaling pathway contains several such proteins that have been implicated in a number of diseases, including a variety of cancers and some psychiatric disorders. To assess whether the activation of this pathway is influenced by genetic factors, we measured phosphorylated and total levels of three key proteins in the pathway (AKT1, p70S6K, 4E-BP1) by ELISA in 122 lymphoblastoid cell lines from 14 families. Interestingly, the phenotypes with the highest proportion of genetic influence were the ratios of phosphorylated to total protein for two of the pathway members: AKT1 and p70S6K. Genomewide linkage analysis suggested several loci of interest for these phenotypes, including a linkage peak for the AKT1 phenotype that contained the *AKT1* gene on chromosome 14. Linkage peaks for the phosphorylated:total protein ratios of AKT1 and p70S6K also overlapped on chromosome 3. We selected and genotyped candidate genes from under the linkage peaks, and several statistically significant associations were found. One polymorphism in *HSP90AA1* was associated with the ratio of phosphorylated to total AKT1, and polymorphisms in *RAF1* and *GRM7* were associated with the ratio of phosphorylated to total p70S6K. These findings, representing the first genomewide search for variants influencing human protein phosphorylation, provide useful information about the PI3K/AKT/mTOR pathway and serve as a valuable proof of concept for studies integrating human genomics and proteomics.

## Introduction

Recent advances in genetics have included large genomewide association studies in which inherited variants have been associated with a particular clinical outcome, such as type 2 diabetes [1]–[3]. However, when there is an understanding of the biological basis of a particular disease, a potentially more tractable approach is to search for variants that influence the physiological hallmarks of the disease [4], [5]. To this end, studies have identified variants associated with biomarkers such as blood glucose levels for metabolic syndrome or bone mineral density for osteoporosis [6]–[10].

On a more molecular level, several groups have published analyses of genetic variation associated with mRNA expression of most human genes, often using cell line models [11]–[13]. These results have been used to identify expression quantitative trait loci (eQTLs) and potential causal variants for additional phenotypes, such as response to drugs or radiation [14]–[16]. The scientific potential of eQTL studies rests on the idea that a gene's mRNA expression level is related to the expression level of its encoded protein, which drives the phenotype in question. However, mRNA expression levels rarely demonstrate strong correlations with protein expression, and loci have been identified for comparatively few human protein levels [17]–[22]. We could not identify any studies that have been clearly focused on identifying genetic influences on the post-translational modifications that can be so critical in determining a protein's activity. Proteins involved in cell signaling are particularly influenced by alterations like phosphorylation, so understanding genetic influences on corresponding mRNA or total protein levels may be largely irrelevant when considering diseases dependent on alterations of signaling.

The phosphoinositide 3-kinase/AKT/mechanistic target of rapamycin (PI3K/AKT/mTOR) signaling pathway is well-characterized and has been linked to the pathology of a number of diseases. The stimulation of this pathway begins with the activation of receptor tyrosine kinases, which promotes the recruitment and activation of PI3K family members [23], [24]. PI3Ks can additionally be activated by signaling mediated by G protein coupled receptors and Ras [25], [26]. Upon activation, PI3Ks phosphorylate phosphotidylinositol-4,5-bisphosphate (PIP_2_) to convert it to phosphotidylinositol-3,4,5-trisphosphate (PIP_3_), a process opposed by PTEN [23], [24]. Increased PIP_3_ levels cause AKT1 to be recruited to the plasma membrane, where it is phosphorylated and activated at Thr308 by PDPK1 and at Ser473 by mTOR complex 2 (mTORC2) [25], [27], [28]. AKT1 then phosphorylates a number of targets, resulting in the activation of mTOR complex 1 (mTORC1), which in turn phosphorylates additional proteins, including p70 S6 kinase (p70S6K) and 4E-BP1 [29]–[31]. The net result of the activation of this pathway is an increase in translation of some proteins, including key growth regulatory proteins like cyclin D1 and MYC [30], [32].

While somatic mutations in the PI3K/AKT/mTOR pathway are common in many types of cancer, inherited variation has also been implicated in cancer and several psychiatric diseases. For example, PI3K/AKT/mTOR signaling is altered in models of schizophrenia, and several single nucleotide polymorphisms (SNPs) in the *AKT1* gene have been associated with the development of the disease as well as with total AKT1 protein levels [33]–[35]. In contrast to these common SNPs, inherited rare variants in other pathway genes, such as *PTEN*, *TSC1* and *TSC2* have long been known to cause cancer syndromes [36]–[38]. The PI3K/AKT/mTOR pathway may also be involved in mediating the response to cancer treatment; one study has claimed associations between SNPs in several pathway genes and several esophageal cancer outcomes including recurrence risk, survival and treatment response [39].

Given the importance of the PI3K/AKT/mTOR pathway in such a broad array of traits and diseases, a better understanding of variation in its activation is essential. Utilizing a commonly used lymphoblastoid cell line collection, we used standard protein and human genetics techniques to conduct the first search for variants that are associated with protein-based measures of PI3K/AKT/mTOR activity. To our knowledge, this also represents the first attempt at identifying variants that influence specific post-translational modifications. Here, we present evidence for the statistically significant associations of several SNPs with measures of PI3K/AKT/mTOR pathway activity.

## Methods

### Cell culture and protein preparation

122 lymphoblastoid cell lines from 14 families in the publicly available CEPH (Centre d'Etude du Polymorphisme Humain) collection were seeded at a density of 250,000 cells/ml and a total volume of 20 ml in duplicate flasks. Variation in cell culture conditions was minimized by thawing all cell lines in a total of four batches, growing all cell lines in the same incubator, using cell lines within a maximum of 2 passages of each other, performing the bulk of the cell culture over a three-week period and using cell culture reagents with identical lot numbers. Growth media consisted of RPMI (Invitrogen) plus 10% fetal bovine serum (Sigma). After 48 hours, contents of duplicate flasks were pooled, and cells were washed twice in ice-cold PBS before being resuspended in lysis buffer (100 mM Tris, 100 mM NaCl, 1 mM EDTA, 1 mM EGTA, 1% Triton-X, 10% glycerol, 0.1% SDS, 0.5% sodium deoxycholate, all chemicals from Fisher) supplemented with 1 mM PMSF (Sigma) and protease and phosphatase inhibitors (Pierce). Cell suspensions were incubated on ice for 30 minutes with periodic vortexing before centrifugation at 13,000×*g* at 4°C for 10 minutes. Supernatants were then removed, applied to Vivaspin 500 concentrating columns (Sartorius) and spun at 15,000×*g* at 4°C for 1 hour. Concentrated lysates were stored at −80°C, and protein concentrations were determined using the Bio-Rad DC Protein Assay. All cell lysates experienced exactly one freeze-thaw cycle.

### ELISAs

ELISAs were performed using kits for specifically measuring phospho-AKT1 (pS473), total AKT1, phospho-p70S6K (pT389), total p70S6K, phospho-4E-BP1 (pT46), and total 4E-BP1 (Invitrogen/Biosource). Each of the 122 protein samples were assayed in triplicate when sufficient protein was available, and recombinant protein standards and pooled lysate standards were placed on each 96-well plate for quality control purposes. All antibody-bound plates for each protein were from a single lot, and additional reagents for each protein were also checked to make sure they came from the same lot. If not, they were pooled before use. Kit protocols were followed exactly with one exception: protein samples were first diluted to achieve equal concentrations before applying equal volumes of lysate to the plates. Final absorbance at 450 nm was read using a Beckman Coulter DTX880 plate reader. Sample information, raw data, standard curves and other information is available upon request.

### Data cleaning, heritability and linkage analysis

For each assay, we confirmed that standard curves were acceptable and that samples fell within the range defined by the standards. Triplicate OD readings were averaged for both the phosphorylated and total protein ELISAs, and ratios of phosphorylated to total protein were calculated by dividing the respective OD values. It should be noted that the use of antibodies and detection reagents of varying concentrations and affinities in the ELISA kits for phosphorylated and total proteins can produce higher OD readings for the phosphorylated form of a protein relative to the OD readings for its total form, resulting in a ratio phenotype greater than 1. This does not imply that there is more phosphorylated protein in the sample than there is total protein. In total, results were obtained for nine measured phenotypes (total protein, phosphorylated protein, and phosphorylated:total protein ratio for AKT1, p70S6K and 4E-BP1). Genetic analyses were conducted using the SOLAR software package. To adjust the data to meet SOLAR's requirements, each of the nine phenotypes was assessed for normality, transformed with an inverse or logarithmic transformation if appropriate, and multiplied by a constant. To assess heritability for these phenotypes, polygenic models were created in SOLAR. A handful of covariates were screened for each phenotype, including age, sex, age × sex, age^2^, age^2^ × sex, ELISA plate number, and protein concentration of the lysate. While initial protein concentration was the only significant covariate for the AKT1 ratio, five variables emerged as significant covariates for the p70S6K ratio. These included two variables describing on which ELISA plate the samples were measured and three variables related to the individual's age at the time the pedigrees were initially obtained (age, age*sex, and age^2^*sex). Since individual ages were not available for many of the CEPH pedigrees, only 62 of 122 individuals were available to use for further analysis of the p70S6K ratio phenotype. The “polygenic –screen” command was used to screen for significant covariates and to estimate the additive genetic heritability.

We conducted multipoint linkage analysis using SOLAR for traits with heritability measures greater than 15%. We obtained genotypes for 126 individuals in 14 CEPH families from version 10.0 of the CEPH genotype database, which includes both microsatellite markers and SNPs. Genetic locations of these markers were determined using data from the Marshfield map. The total number of genotypes available for these markers was 95,278 at 7,403 loci. SOLAR identified 30 markers with Mendelian inconsistencies or other errors, and these were removed. Before running the linkage analysis, we used the “lodadj –calc” command to create a distribution of LOD scores under the null hypothesis of no linkage. We also conducted bivariate linkage analysis of the AKT and p70S6K phosphorylation ratios in SOLAR. For this analysis, covariates significant for each of the individual traits were applied to the respective traits, and testing was performed using the “polygenic –testrhog” command.

### Candidate gene selection

Candidate genes for association analysis were selected using CANDID, a candidate gene identification algorithm [40]. Inputs to CANDID included unaltered output files from the linkage analyses and a list of keywords reflecting existing knowledge about the signaling pathway. Weights for CANDID were set as 1 for literature, 0.2 for protein domains, 0.2 for conservation, and 0.8 for protein-protein interactions, and version 5 of the CANDID databases was used. These weights were selected *a priori* based on the nature of the phenotype under study. The literature criterion was given the highest weight in accordance with the finding in the original CANDID publication that it was by far the most useful of all CANDID criteria. Protein domains and protein-protein interactions were given higher weights than those suggested in the CANDID publication since it seemed likely that causal genes for these cell signaling phenotypes would encode proteins that possess defined functional domains and that are known to interact with some of the proteins under study here. Finally, conservation was weighted slightly higher here as well due to the fact that this signaling pathway is conserved across many species. The list of keywords input to CANDID was: AKT, signaling, phosphatase, kinase, mTOR, PI3K, phosphatidyl, phosphoinositide, p70S6K and AKT1. Several of CANDID's top-ranked genes were selected for further analysis.

### SNP selection, genotyping and association analysis

A total of 86 tag SNPs for six genes (*HSP90AA1*, *KAT2B, RAB5A, RAF1, VHL* and *GRM7*) were selected using an r^2^ cutoff of 0.8. For *AKT1*, a more in-depth SNP selection process was undertaken in which 51 coding SNPs or common intronic or perigenic SNPs were selected from dbSNP. SNP genotyping was performed using the Sequenom platform and DNA that had been extracted from cell lines from the 14 CEPH families. PCR and extension primers were designed using Sequenom AssayDesign software (Table S1). PCR reactions, shrimp alkaline phosphatase digestion, and extension reactions were performed according to Sequenom's standard protocol with one exception: a linear adjustment to the PCR primer concentrations was made in an attempt to standardize mass spectrometer peak heights.

Any SNP or individual that had less than an 85% success rate was removed from further analysis. The Pedstats and MERLIN software packages were also used to identify and remove unlikely genotypes and genotypes that produced Mendelian errors. Invariant SNPs were also removed. The remaining SNPs were tested for Hardy-Weinberg equilibrium in the unrelated individuals. SNPs that passed this test were analyzed in Haploview, and several more SNPs were eliminated as having been tagged by other genotyped SNPs. Genotypes for the remaining SNPs were coded for analysis under an additive (genotypic) model, a dominant model, and a recessive model. Association tests were conducted in SAS using PROC MIXED, with pedigree ID as a class and subject variable and compound symmetry as the covariance structure. Significant SNPs were visualized in Haploview using data from the HapMap CEU population (Version 2, Release 24).

## Results

Three proteins (AKT1, p70S6K and 4E-BP1) involved in PI3K/AKT/mTOR signaling were assayed for this study. We initially selected these proteins due to their robust and reliably detectable expression levels in the cell line model used here as well as their varying positions in the PI3K/AKT/mTOR signaling cascade. Initial studies indicated that the ELISA assays used showed little variation across protein samples experiencing different numbers of freeze-thaw cycles and across protein samples for the same cell line harvested from different cell culture flasks (S1A, S1B). ELISA assays performed on different days using identical protein samples gave slightly different results but generally preserved similar relationships between cell lines (Figure S1C). When experiments measuring two protein phenotypes were completely redone from start to finish for a subset of cell lines, one phenotype still showed a good correlation between replicates (r^2^ = 0.52), while the other did not (Figure S1D, S1E). A strong correlation was not necessarily expected in these cases, however, since cell line and reagent availability issues forced several key differences between the replication experiment and the original, including differing passage numbers for many cell lines and differing reagent lots for the ELISAs. As mentioned above, all possible precautions were taken to insure that samples used to collect data points for linkage and association studies were treated as uniformly as possible from cell culture to ELISA in order to minimize any potential quality control issues.

Each of the three measured proteins displayed variability in the levels of phosphorylated and total protein across the 14 families used in this study. These measurements, as well as the derived ratios of phosphorylated to total protein (calculated using the OD readings for these two ELISAs), varied to different extents. For example, p70S6K showed the lowest fold difference in total protein (1.52-fold), while 4E-BP1 had the highest (2.43-fold). The fold differences for measurements of phosphorylated proteins appeared to be slightly more variable (1.99, 1.35 and 4.80 for AKT1, p70S6K and 4E-BP1, respectively), as did the phosphorylated:total ratios (2.91, 1.46 and 2.92 for AKT1, p70S6K and 4E-BP1, respectively). Plots showing these measurements in unrelated individuals can be found in Figure S2.

Given that variation existed in these nine phenotypes (phosphorylated protein, total protein and phosphorylated:total protein for AKT1, p70S6K and 4E-BP1), we were able to determine the phenotypes' heritabilities, or the percentage of variation in the traits that can be attributed to genetic effects. We assessed normalized phenotypes for relationships with any potential covariates, including age, sex and experimental variables. Heritabilities of the covariate-adjusted phenotypes were generally very low (<3%) with two exceptions: the phosphorylated:total protein ratios for two proteins, AKT1 and p70S6K, displayed moderate heritability (Table 1, *h^2^* = 17.99% and 23.13%, respectively). The p-value for the heritability of the AKT1 ratio was significant (*P* = 0.03, standard error = 0.13), but the p-value for the p70S6K ratio was not (*P* = 0.2, standard error = 0.37), possibly due to a reduced sample size for this phenotype. Age was determined to be a significant covariate for the p70S6K ratio, but age information was only available for 62 individuals, so the heritability and linkage analyses was limited to only these samples.

**Table 1:** Heritabilities of AKT1, p70S6K and 4E-BP1 phenotypes.

Protein	Phenotype	Heritability (%)
AKT	total	2.64
	phosphorylated	2.04
	phosphorylated:total	17.99
p70S6K	total	2.81
	phosphorylated	0
	phosphorylated:total	23.13
4E-BP1	total	2.10
	phosphorylated	0
	phosphorylated:total	0

**Figure 1:**
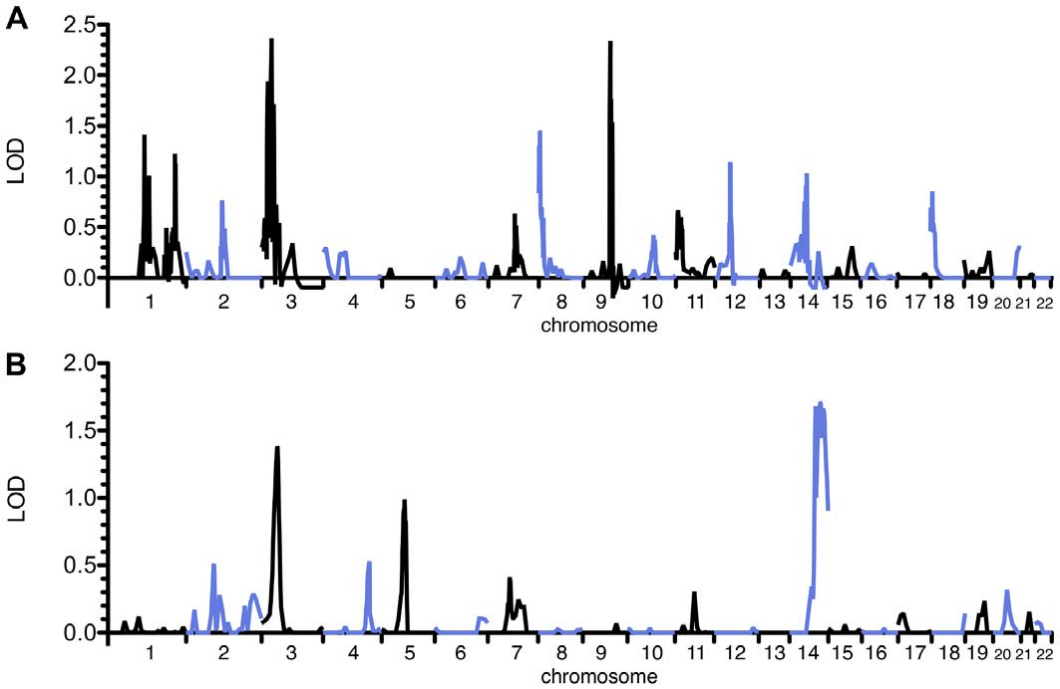
Genomewide linkage analysis results for AKT1 and p70S6K ratio phenotypes. Genomewide linkage analysis results are shown for the ratio of phosphorylated to total p70S6K (A) and AKT1 (B). Chromosomes 1–22 are depicted from left to right, with alternating colors indicating different chromosomes; cM positions across each chromosome increase from left to right. The maximum LOD score for the p70S6K ratio trait is 2.37 and is found on chromosome 3, while the maximum LOD score for the AKT1 ratio is 1.70 on chromosome 14.

**Figure 2:**
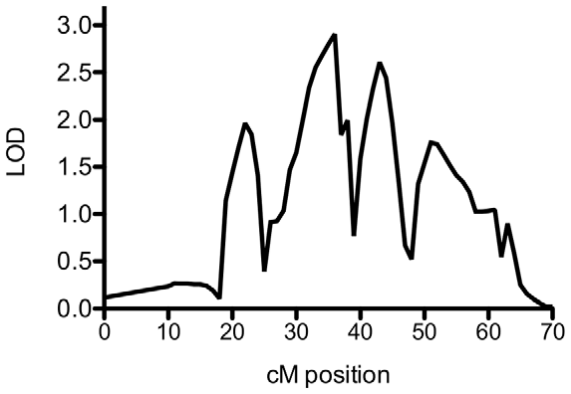
Linkage to chromosome 3 of correlated AKT1 and p70S6K ratio phenotypes. We found the AKT1 and p70S6K ratio traits to be genetically correlated, and we used their shared genetic component for genomewide linkage analysis. A section of chromosome 3 containing the maximum LOD score of 2.91 is shown here.

Since our sample sizes were relatively small and the heritabilities for these two traits were moderate, we lacked the statistical power necessary to conduct a genomewide association study of variants influencing these traits. Instead, we elected to take a three-step approach in which we first conducted conventional genomewide linkage analyses to identify loci of interest, then identified strong candidate genes in those regions, then tested specific variants in those genes. The initial linkage analysis for the AKT1 and p70S6K ratio traits failed to produce genome-wide significant LOD scores, but there was suggestive linkage for p70S6K on chromosome 3, with a maximum LOD score of 2.37 (Figure 1). There appeared to be a second peak on chromosome 9, but the presence of multiple software convergence errors in this region cast some doubt on its reliability. For the AKT1 ratio phenotype, the maximum LOD score was 1.70 on chromosome 14 (Figure 1). Interestingly, there was also a smaller peak for the AKT1 ratio on chromosome 3 (LOD = 1.38 at 57 cM) close to the major peak for the p70S6K ratio at 36 cM. Since AKT1 is known to act upstream from p70S6K in the PI3K/AKT/mTOR pathway, it was possible that the two peaks resulted from genetic variation in this region that influenced one protein's phosphorylated:total protein ratio, which then influenced the other protein's ratio. A bivariate analysis revealed that the phenotypes were genetically correlated (RhoG  = 0.980, *P* = 0.009). A linkage scan for the correlated traits identified a peak on chromosome 3 with a maximum LOD score of 2.91, indicating that genes in this region likely influence both traits (Figure 2). Aside from a peak on chromosome 9 in a region with many convergence errors, no other peak exceeded a LOD score of 2 in this genomewide scan (Figure S3). We next incorporated each of the ratio traits as a covariate for the other trait. This analysis resulted in retention of the peak for the AKT1 ratio but not for the p70S6K ratio (data not shown). This implied that the causal variants in this region mainly influence the AKT1 ratio, which in turn influences the p70S6K ratio.

To search for potential positional and biological candidate genes, we selected two regions containing linkage peaks: a region from 87 cM to the q-terminus on chromosome 14 corresponding to the AKT1 ratio peak, and a region from 19.5 cM to 54 cM on chromosome 3 corresponding to the linkage peak for the correlated AKT1 and p70S6K ratio traits. We then chose keywords related to cell signaling and the PI3K/AKT/mTOR pathway and entered them, along with the linkage data and other information, into an analysis using CANDID, a candidate gene identification algorithm [40]. CANDID scored and ranked all genes within the defined regions, and the top ten genes for each peak are shown in Table 2. The *AKT1* gene represented an exceptionally strong candidate for the AKT1 ratio peak on chromosome 14, while the top-ranked genes on chromosome 3 included *RAF1*, *VHL*, and *RAB5A*, which are signaling proteins in pathways that interact with PI3K/AKT/mTOR signaling (Figure S4).

**Table 2:** Top ten identified candidate genes for the AKT1 and p70S6K phosphorylated:total protein ratio phenotypes.

Rank	Chromosome 14 gene symbols(CANDID score)	Chromosome 3 gene symbols(CANDID score)
1	*AKT1* (55.5)	*RAF1* (69.4)
2	*HSP90AA1* (54.2)	*VHL* (20.6)
3	*CALM1* (33.6)	*KAT2B* (16.7)
4	*TRAF3* (17.2)	*GRM7* (11.2)
5	*EVL* (12.2)	*RAB5A* (11.4)
6	*TSHR* (9.9)	*THRB* (9.7)
7	*MTA1* (9.3)	*IRAK2* (8.4)
8	*BEGAIN* (9.1)	*ATP2B2* (7.7)
9	*CCNK* (8.7)	*SH3BP5* (7.5)
10	*YY1* (7.9)	*PPARG* (7.2)

We selected the top two genes under the chromosome 14 peak (*AKT1* and *HSP90AA1*) and the top five genes under the chromosome 3 peak (*RAF1*, *VHL*, *KAT2B*, *GRM7* and *RAB5A*) to test whether variants in these genes are associated with either the AKT1 phosphorylated:total protein ratio or the p70S6K phosphorylated:total protein ratio. Since the *AKT1* gene was such a strong candidate, 51 SNPs in or near *AKT1* were genotyped along with an additional 7 tag SNPs from *HSP90AA1.* One of these SNPs, rs1190584, was significantly associated with the AKT1 ratio phenotype (Table 3, Table S2). *P*-values for this SNP were 0.0036 under a recessive model and 0.0042 under an additive model. Though this SNP lies approximately 62.5 kilobases upstream of *HSP90AA1* in an intron for *WDR20*, it is part of a block of linkage disequilibrium that extends into the 5′ end of the *HSP90AA1* gene (Figure S5).

**Table 3:** SNPs significantly associated with phosphorylated:total AKT1 ratio or phosphorylated:total p70S6K ratio.

Phenotype	SNP	Gene symbol	Additive model	Dominant model	Recessive model
AKT1 ratio	rs1190584	*HSP90AA1*	0.0042*	0.564	0.0036*
p70S6K ratio	rs12630300	*GRM7*	4.10×10^−4^ *	-	4.10×10^−4^ *
p70S6K ratio	rs5746223	*RAF1*	6.35×10^−5^ **	6.35×10^−5^ **	-
p70S6K ratio	rs713178	*RAF1*	4.57×10^−4^ *	5.65×10^−4^ *	0.356
p70S6K ratio	rs9855183	*RAF1*	6.35×10^−5^ **	6.35×10^−5^ **	-

*Significant using false discovery rate of 5%.

**Significant at Bonferroni-corrected threshold.

We tested the SNPs on chromosome 3 for association with the phosphorylated:total p70S6K ratio because its linkage evidence in this region was stronger than that of the phosphorylated:total AKT1 ratio. Out of 75 tested SNPs, four showed significant association under at least one model. Three of these (rs713178, rs5746223 and rs9855183) are distributed across a 25-kilobase region that includes *RAF1* and are in moderately high linkage disequilibrium with each other (Figure S5). Of the three, rs5746223 is the only SNP located in the *RAF1* gene – rs713178 and rs9855183 are both located downstream of *RAF1*, in introns of the *MKRN2* gene. The fourth significant SNP, rs12630300, is in the intron of the *GRM7* gene and is separated from the *RAF1* SNPs by approximately 6 megabases. These four SNPs were highly significant, with *P*-values ranging from 4.10×10^−4^ to 6.35×10^−5^. No other SNPs approached significant association with this phenotype.

## Discussion

Variation in the activation of the PI3K/AKT/mTOR pathway has been linked to a variety of human diseases. In an *in vitro* model using lymphoblastoid cell lines from the Centre d'Etude du Polymorphisme Humain (CEPH) collection, we confirmed variation in the amounts of total and phosphorylated AKT1, p70S6K and 4E-BP1. The degree of genetic influence on the phosphorylation of these three proteins appears to be similar to analogous results using gene expression and protein phenotypes. In cell lines from the CEPH collection, approximately 30% of differentially expressed genes appear to have significant heritability, and heritability is high (greater than 60%) for only 10% of these genes [5], [12], [41]. In contrast to the wealth of expression data available, information on protein expression is more scarce. Handfuls of proteins have been measured in blood samples, including C-reactive protein, serum insulin and fibrinogen, and heritabilities for these traits range from 18–60% [42], [43]. Heritability data for protein phenotypes in human cell lines is even scarcer. One study was identified in which the heritability of a specific protein (chromogranin B) was determined, and the results were highly variable and dependent on which cleavage product was measured (*h^2^* = 0.378–0.910) [44]. A more recent study examined 544 protein phenotypes in 24 cell lines and found only 24 of these phenotypes to have an estimated heritability greater than 50% [21].

Given that relatively few gene and protein expression phenotypes are strongly heritable, the finding presented here of low heritability for the total levels of AKT1, p70S6K and 4E-BP1 is not surprising. Additionally, the two heritability levels observed for the ratios of phosphorylated to total AKT1 and p70S6K (17.99% and 23.13%, respectively) fall within the range of *h^2^* values observed for other gene and protein expression phenotypes. It is also interesting to note that both of the heritable phenotypes identified in this study are ratios. It is possible that by taking a ratio of two measurements, some experimental sample-to-sample noise is eliminated, reducing the overall variation and increasing the heritability. In fact, a study of metabolic phenotypes identified stronger genetic associations when considering ratios of metabolites rather than single metabolite measurements [45]. Intriguingly, total AKT levels were not very heritable at all (2.64%) in this study (Table 1). Since this trait does not appear to be genetically influenced, it is unlikely that this dataset would confirm the associations between *AKT1* SNPs and total protein levels reported by other groups [33]–[35]. This could reflect differences in experimental platforms (cell lines vs. tissue samples) or detection methods (immunoblotting vs. ELISAs).

The two phenotypes with moderate heritabilities produced interesting, though not statistically significant, linkage analysis results. In general, it may be that their lower LOD scores are reflective of the small sample size used in these experiments; the use of additional CEPH pedigrees might have increased the LOD score of these peaks. The secondary peak for the AKT1 phenotype on chromosome 3 initially seemed to be too low to accept statistically. However, when it was considered in tandem with the p70S6K phenotype that mapped to the same area, the traits were found to share a genetic component that produced a much higher joint LOD score of 2.91 in the same area. Given what is known about the architecture of the PI3K/AKT/mTOR signaling pathway, this result is highly plausible biologically. A variant in this region that significantly influences the ratio of phosphorylated to total AKT1 would likely also produce a change in the ratio of phosphorylated to total p70S6K, since AKT1 activation results in the mTOR-mediated phosphorylation and activation of p70S6K. The disappearance of the chromosome 3 linkage peak for p70S6K when AKT1 levels are included as a covariate confirms this directionality. This represents evidence that this cell-based platform for genetic analysis can confirm existing biology as well as be used to uncover new information.

Through association tests, we identified potentially novel roles for several genes known to influence PI3K/AKT/mTOR signaling. HSP90, the protein product of *HSP90AA1*, has long been known to affect signaling by regulating the stability of a variety of proteins, including AKT1 [46], [47]. As such, it is a popular target for recent oncology drug development efforts [48], [49]. However, little is known about natural variation within the *HSP90AA1* gene and its effects on the activity of HSP90. Additional investigation of the *HSP90AA1* SNP that was significant in this study, rs1190584, is necessary in order to identify the mechanism by which a potential variant in the *HSP90AA1* region affects the ratio of phosphorylated to total AKT1. Since AKT1 must be localized at the plasma membrane in order to be phosphorylated at residue S473, possible mechanisms include methods by which altered HSP90 activity might inhibit this localization. If a causal variant in this region is found to work by altering the activity or overall levels of HSP90, it may influence patient response to the new HSP90 inhibitors and could be considered for future pharmacogenomic studies.

The *RAF1* gene encodes RAF1 (also known as c-RAF), a key member of the mitogen-activated protein kinase (MAPK) signaling cascade. Though the PI3K/AKT/mTOR and MAPK pathways are usually thought of as distinct, cross-talk between them has been documented in a variety of contexts [50]–[52]. However, reports describing the mechanism for this cross-talk are contradictory. If a functional variant in *RAF1* can be shown to influence the ratio of phosphorylated to total AKT1, its method of influencing *RAF1* expression levels or RAF1 protein structure could help elucidate the mechanism for PI3K/AKT/mTOR-MAPK cross-talk. As with HSP90, RAF1 represents a target for oncology drug discovery, and additional information gained about its mechanism could assist with the development or testing of new treatments. A clearly necessary first step in exploring these associations is to confirm them in another cell line collection.

Though cell line collections have proven quite useful in work conducted by our group and others, there are obvious limitations to their use. Cell lines are convenient to work with but cannot substitute for freshly obtained tissue samples in terms of their ability to reflect real physiology. There are also potential issues with cell lines transformed using Epstein Barr virus (EBV), like those used for this work. The viral integration sites are not known for these cell lines and in some cases may have caused aberrant activation or inhibition of nearby genes. In this scenario, variation in DNA due to EBV integration could well be influencing the traits measured here, but since this is not inherited, it would manifest as environmental variation, lowering the resulting heritability estimates. Cell lines also behave differently as they go through an increasing number of passages, so careful attempts must be made to utilize cell lines of similar passage number and thereby minimize environmental variation. Variation in culture media and supplements should also be minimized by using reagents from identical lots. For cell signaling phenotypes like those measured here, this is especially important, since serum concentration and culture conditions can affect these phenotypes. If heritability estimates are decreased sufficiently by these types of effects, some phenotypes that are truly influenced by inherited variation may be erroneously dismissed as non-genetic. Regardless of these caveats and in the absence of more convenient platforms, cell culture work continues to produce intriguing results that should be investigated more closely using other techniques.

The work presented here represents a unique and successful genomic method for exploring inter-individual variation in cell signaling. With high-throughput platforms for measuring protein levels and post-translational modifications on the horizon, this method will be a simple, straightforward way in which to identify biologically significant variants that may be relevant to any field in which cell signaling is important, including cancer and psychiatric disease research.

## Supporting Information

Figure S1 **Sources of biological and technical variation in ELISA assay results.** The protein phenotypes measured by ELISA varied little after several freeze-thaw cycles, only beginning to vary after 4 cycles (representative data shown in A). Likewise, there was little variation in measurements produced when cell lines were split across multiple flasks and processed separately through the ELISA measurements (representative data shown in B). ELISA assays performed on different days tended to give slightly different readings (C). However, for linkage and association studies, it is most essential that the relationships between samples remain preserved, and this was generally seen (representative data shown in C). A complete replication of the ELISA experiments for the phospho-AKT/total AKT (D) and phospho-p70S6K/total p70S6K ratios (E) was undertaken in a subset of cell lines. For this replication, many cell lines were grown at a different passage number than they were originally. Additionally, the ELISA kits used for this replication were of different lot numbers than those used for the original experiment (a known source of across-experiment variation). Despite these differences, the AKT ratio replicated well, but the p70S6K ratio did not.
(TIFF)

Figure S2 **Variation in AKT1, p70S6K and 4E-BP1 phenotypes.** Measurements of three different proteins in cell lines from 26 unrelated individuals are shown here. The proteins are AKT1 (top row), p70S6K (middle row) and 4E-BP1 (bottom row). The measurements are of total amounts of the protein (left column), of the amounts of the protein phosphorylated at the site indicated (middle column) and of the ratio of phosphorylated to total protein (right column). Measurements were adjusted for significant covariates before graphing and are sorted from highest to lowest within each graph. Error bars represent standard error of the mean (s.e.m.).
(TIF)

Figure S3 **Genomewide linkage analysis results of correlated AKT1 and p70S6K ratio phenotypes.** The maximum LOD score of 2.91 is on chromosome 3. The peak on chromosome 9 is believed to be an artifact due to the presence of multiple errors in that region.
(TIFF)

Figure S4 **Candidate gene locations within linkage peaks on chromosomes 3 and 14.** The LOD peak on chromosome 3 for the correlated ratios of phosphorylated to total AKT and p70S6K is shown in (A), and the LOD peak on chromosome 14 for the ratio of phosphorylated to total AKT1 is shown in (B). The locations of the selected candidate genes for each trait are depicted by vertical lines in the corresponding panel. The chromosome 3 candidate genes, from left to right, are *GRM7* (23.89 cM), *VHL* (30.72 cM), *RAF1* (36.89 cM), *RAB5A* (43.07 cM) and *KAT2B* (43.19 cM). The chromosome 14 genes are *HSP90AA1* (131.52 cM) and *AKT1* (134.3 cM). The cM values were determined using CANDID and were calculated for each gene by using the gene's midpoint (in base pairs) to interpolate its genetic location using two adjacent Marshfield map markers with known physical locations.
(TIF)

Figure S5 **Linkage disequilibrium surrounding significant SNPs.** Haploview plots showing the extent of linkage disequilibrium surrounding significant SNPs in *HSP90AA1* (A) and *RAF1* (B) are shown here. The physical locations of the SNPs in relation to their associated genes is also depicted.
(TIFF)

Table S1 **SNP genotyping primer information.**
(DOC)

Table S2 **All association test results for SNPs on chromosomes 14 and 3.**
(DOC)
