# Supplementary figures and images for: Genomewide Analysis of Inherited Variation Associated with Phosphorylation of PI3K/AKT/mTOR Signaling Proteins
Source: PLoS One. 2011 Sep 19;6(9):e24873. doi: 10.1371/journal.pone.0024873 (PMC3176272; doi:10.1371/journal.pone.0024873)

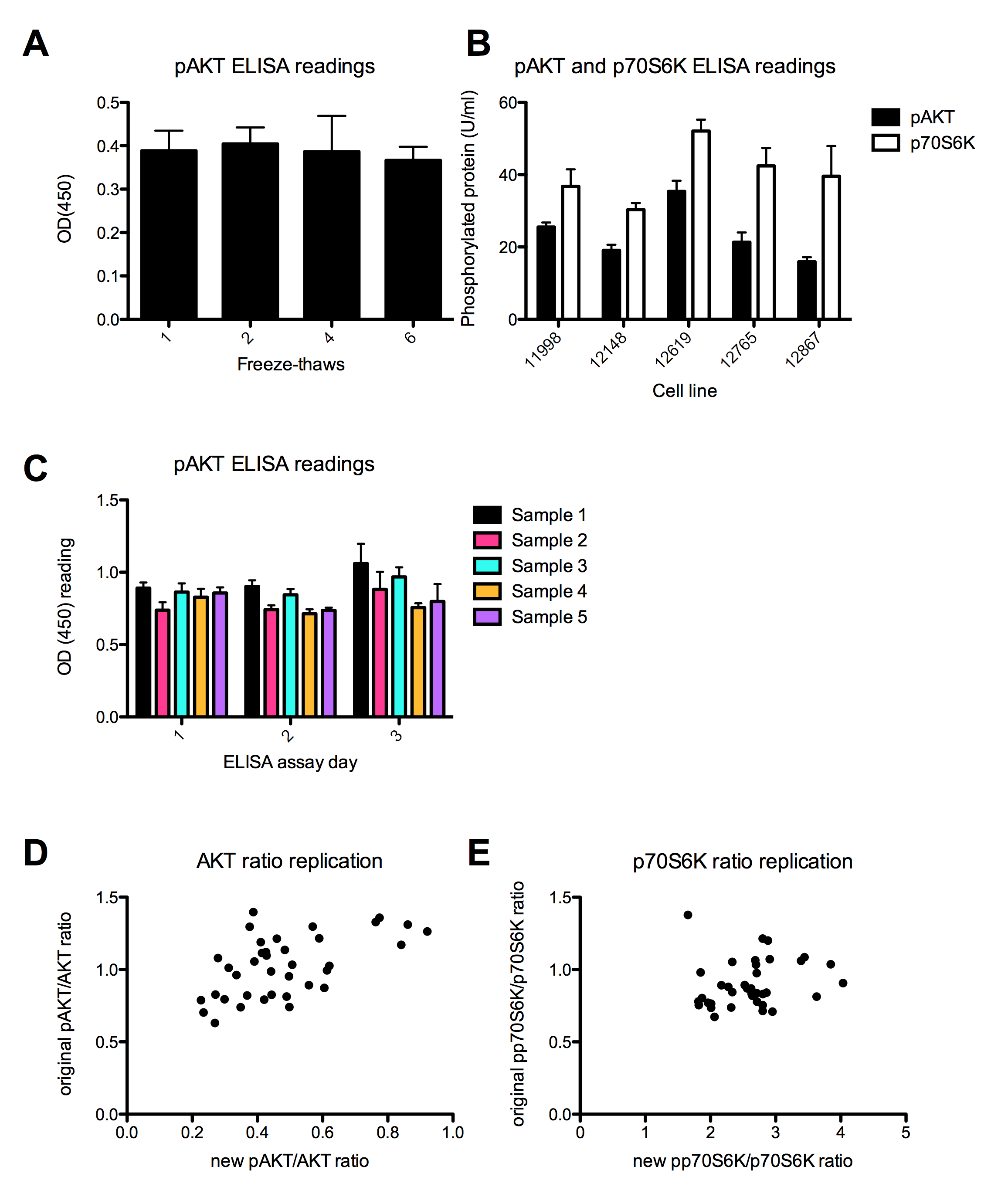

Supplement: Figure S1 — Sources of biological and technical variation in ELISA assay results. The protein phenotypes measured by ELISA varied little after several freeze-thaw cycles, only beginning to vary after 4 cycles (representative data shown in A). Likewise, there was little variation in measurements produced when cell lines were split across multiple flasks and processed separately through the ELISA measurements (representative data shown in B). ELISA assays performed on different days tended to give slightly different readings (C). However, for linkage and association studies, it is most essential that the relationships between samples remain preserved, and this was generally seen (representative data shown in C). A complete replication of the ELISA experiments for the phospho-AKT/total AKT (D) and phospho-p70S6K/total p70S6K ratios (E) was undertaken in a subset of cell lines. For this replication, many cell lines were grown at a different passage number than they were originally. Additionally, the ELISA kits used for this replication were of different lot numbers than those used for the original experiment (a known source of across-experiment variation). Despite these differences, the AKT ratio replicated well, but the p70S6K ratio did not. (TIFF) [file pone.0024873.s001.tiff]

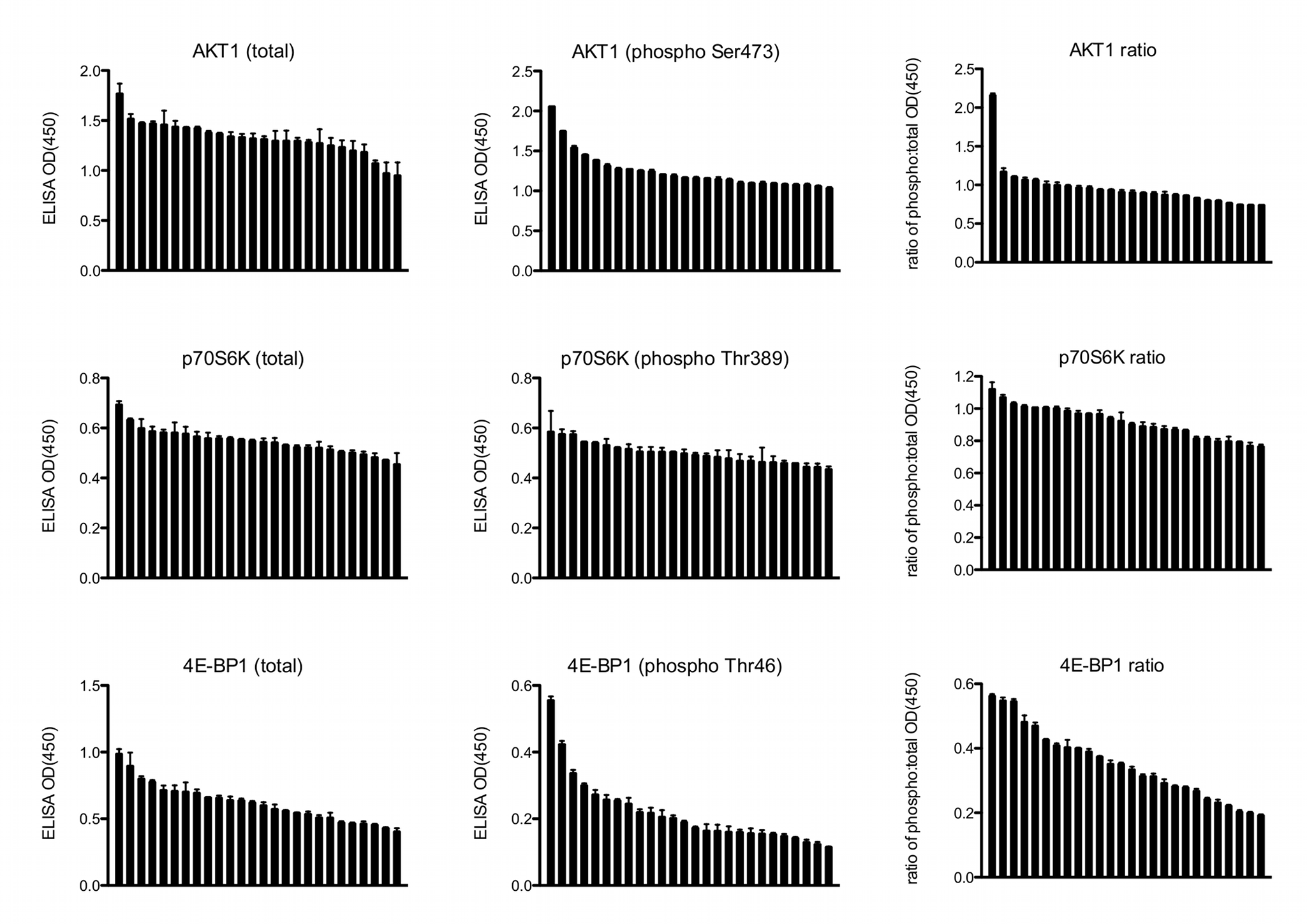

Supplement: Figure S2 — Variation in AKT1, p70S6K and 4E-BP1 phenotypes. Measurements of three different proteins in cell lines from 26 unrelated individuals are shown here. The proteins are AKT1 (top row), p70S6K (middle row) and 4E-BP1 (bottom row). The measurements are of total amounts of the protein (left column), of the amounts of the protein phosphorylated at the site indicated (middle column) and of the ratio of phosphorylated to total protein (right column). Measurements were adjusted for significant covariates before graphing and are sorted from highest to lowest within each graph. Error bars represent standard error of the mean (s.e.m.). (TIF) [file pone.0024873.s002.tif]

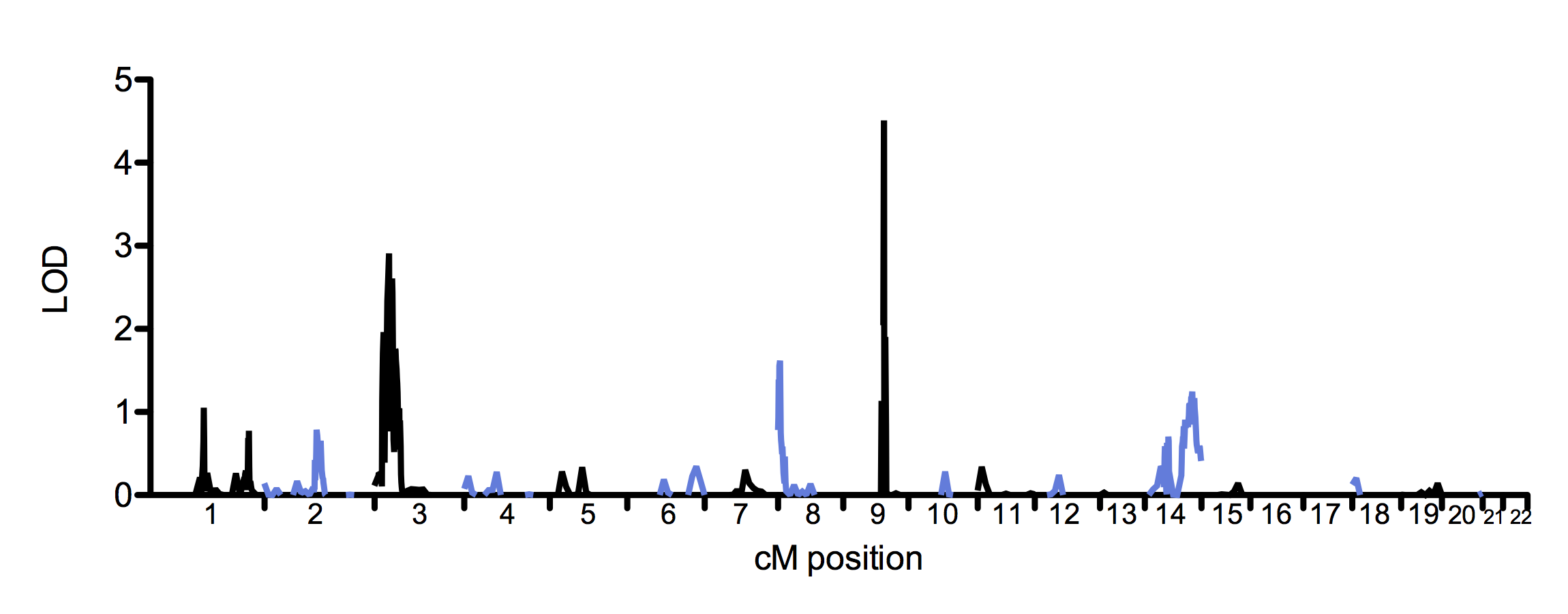

Supplement: Figure S3 — Genomewide linkage analysis results of correlated AKT1 and p70S6K ratio phenotypes. The maximum LOD score of 2.91 is on chromosome 3. The peak on chromosome 9 is believed to be an artifact due to the presence of multiple errors in that region. (TIFF) [file pone.0024873.s003.tiff]

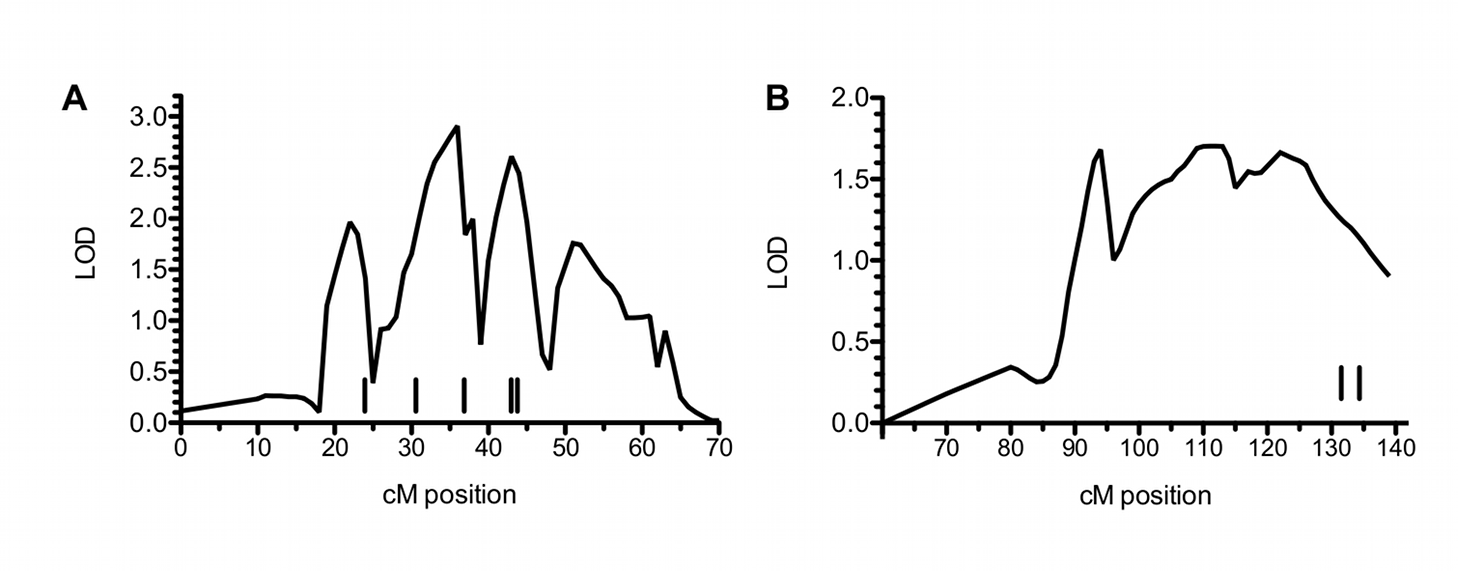

Supplement: Figure S4 — Candidate gene locations within linkage peaks on chromosomes 3 and 14. The LOD peak on chromosome 3 for the correlated ratios of phosphorylated to total AKT and p70S6K is shown in (A), and the LOD peak on chromosome 14 for the ratio of phosphorylated to total AKT1 is shown in (B). The locations of the selected candidate genes for each trait are depicted by vertical lines in the corresponding panel. The chromosome 3 candidate genes, from left to right, are GRM7 (23.89 cM), VHL (30.72 cM), RAF1 (36.89 cM), RAB5A (43.07 cM) and KAT2B (43.19 cM). The chromosome 14 genes are HSP90AA1 (131.52 cM) and AKT1 (134.3 cM). The cM values were determined using CANDID and were calculated for each gene by using the gene's midpoint (in base pairs) to interpolate its genetic location using two adjacent Marshfield map markers with known physical locations. (TIF) [file pone.0024873.s004.tif]

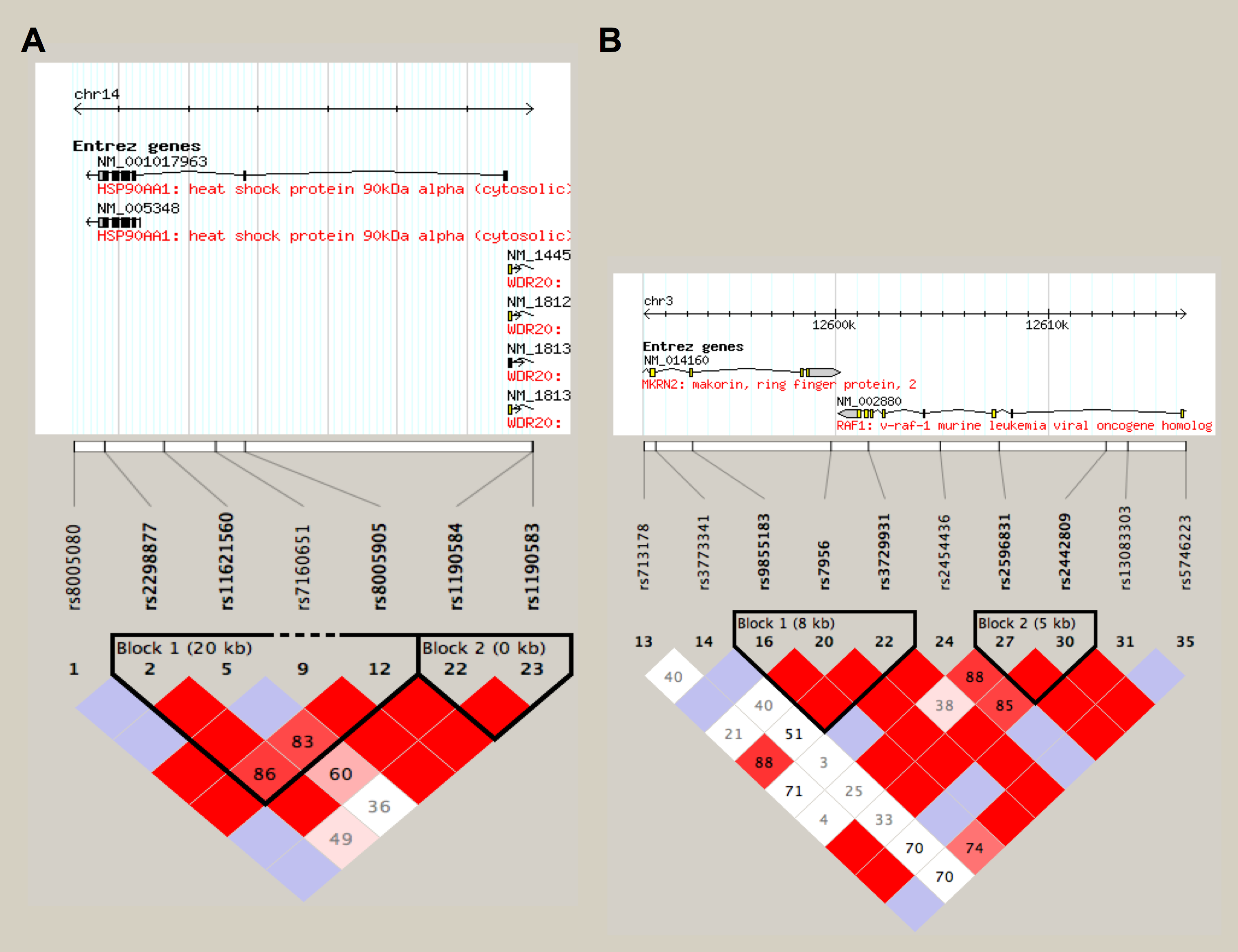

Supplement: Figure S5 — Linkage disequilibrium surrounding significant SNPs. Haploview plots showing the extent of linkage disequilibrium surrounding significant SNPs in HSP90AA1 (A) and RAF1 (B) are shown here. The physical locations of the SNPs in relation to their associated genes is also depicted. (TIFF) [file pone.0024873.s005.tiff]
