# Supplementary material for: Genomewide Analysis of Inherited Variation Associated with Phosphorylation of PI3K/AKT/mTOR Signaling Proteins
Source: PLoS One. 2011 Sep 19;6(9):e24873. doi: 10.1371/journal.pone.0024873 (PMC3176272; doi:10.1371/journal.pone.0024873)
Supplement: Table S1 — SNP genotyping primer information. (DOC) [file pone.0024873.s006.doc]

| **Table S1: SNP genotyping primer information** | | | | | |
| --- | --- | --- | --- | --- | --- |
| Assay | SNP | Gene | PCR primer 1 (ACGTTGGATG-) | PCR primer 2 (ACGTTGGATG-) | Extension primer |
| 1 | rs1130214 | *AKT1* | AAGACAGGACCAGGATGCAG | TGGGGTTTCTCCCAGGAGG | GGGTTTCTCCCAGGAGGTTTTTG |
| 1 | rs1130233 | *AKT1* | CAGCTGTTCTTCCACCTGTC | ACACAATCTCAGCGCCATAG | TGGCCCGGTCCTCGGAGAACACACG |
| 1 | rs1130295 | *AKT1* | TGTCTCTGAGGACGTCATCG | TAAACCTTGCTCCTCTGTCC | GACTCCCCCATCCCTGGTCCCATCCCA |
| 1 | rs1130443 | *AKT1* | TACCGTGGAGAGATCATCTG | TTTGTGACAGGAAAGCCCTC | CCCTCCCCCTTCCCCTT |
| 1 | rs11555432 | *AKT1* | TCTCCTCCATGAGGATGAGC | ATGTACGAGATGATGTGCGG | ACCAGGACCATGAGAAGC |
| 1 | rs11555434 | *AKT1* | CGACACTGTGGCCTTGTTTC | AAGCGATGCTGCATGATCTC | TTGCGTCCTCGGAGCCCCC |
| 1 | rs11848899 | *AKT1* | GCAGTTGGTGGATAAGATGG | ACGCTAACTGAGCGGGAAGA | AGCGGGAAGACTGATGA |
| 1 | rs12588965 | *AKT1* | GTTTCTGTCGCTGGCCCTA | AGGACAGATGTGCCTGGGAT | GTGGCAGGCGCGGTACGGGAGC |
| 1 | rs17846831 | *AKT1* | ACAGCATTGCGTGTGCTCAG | TTTTGCGGCACACCTGAGTA | TGCGGCACACCTGAGTACCTGGC |
| 1 | rs2230508 | *AKT1* | GACTACCTGCACTCGGAGAA | TCAGTGCCGCCAGGCCCCCA | ACCTTGAGGTCCCGGTACAC |
| 1 | rs2494731 | *AKT1* | TGCAGACAGGACTCTCAATG | TCACGTGTGCACATCACCTT | TCTCACCTTATAGTCACCCTT |
| 1 | rs34558581 | *AKT1* | CAATGCCACATTGCGCATAG | CCTCAGCCCTCAGAACAATC | CCTAGCCCTCAGAACAATCCGATTCA |
| 1 | rs34670300 | *AKT1* | CACCCTCATCTCCACCCTG | ACGCCATGAAGATCCTCAAG | GATCCTCAAGAAGGAAGTCAT |
| 1 | rs35416681 | *AKT1* | TGCCACCAGGTTGAACTGAG | ATGTGTCCCCTCACTCTGTC | CCCGGCTGTCTGTCACCAGCTA |
| 1 | rs36214921 | *AKT1* | ACTTTTCGTCCGTGGGAAAC | TGCCCCGGGGTTGGAGAAA | GGGTTGGAGAAAGACTCGC |
| 1 | rs3803304 | *AKT1* | TATCAGTGTAGTCTGGGAGG | TACACCTCCATCCCCTCATC | GCATCCCCAGGCTGCACCTGCCCCT |
| 1 | rs3840005 | *AKT1* | ACAGGGAGTCAGGGAGGGC | AGATGATCTCTCCACGGTAG | CACTTGACCTTTTCGAC |
| 2 | rs10138227 | *AKT1* | CCAGGAGGTTTTTGGGCTTG | AGGAAGACAGGACCAGGATG | GTGCAGGCCACTGGCGCAAA |
| 2 | rs1130526 | *AKT1* | ATAATATGGAACCTTCCCTC | CACTCTTCCACCCAGCAAAG | TTGAAAAGCAACTTTTATTGAA |
| 2 | rs11555430 | *AKT1* | TGGCTTCTCTCAAATGCACC | ACGATAGCTTGGAGGGATGG | TATAGCTTGGAGGGATGGAGAGGCG |
| 2 | rs11555433’ | *AKT1* | TTGAGTACCTGAAGCTGCTG | GATCTTCATGGCGTAGTAGC | CTGTGGCCTTCTCCTTC |
| 2 | rs11555435 | *AKT1* | TGGCCGAGTAGGAGAACTG | TCTACACCCACAGATGACAG | CCACAGATGACAGCATGGAGTGT |
| 2 | rs12881616 | *AKT1* | ACAGCATTGCGTGTGCTCAG | TTTTGCGGCACACCTGAGTA | TGAGTACCTGGCCCCCG |
| 2 | rs17846825 | *AKT1* | CAGGCACAGGCAGAAGTGG | GCTGAAGAGATGGAGGTGTC | GTCCCTGGCCAAGCCCAAGCACCG |
| 2 | rs2494746 | *AKT1* | AGCTTCTGGCTCTGCTTCC | AACAAGCAGGGGACAGCACA | GGGATGGAGAAGGCAGGATG |
| 2 | rs2494748 | *AKT1* | ACAGGCACTCACAGACCCT | ACAAACGAGGTTAGTACCCG | TGCCTGGGGAGGGAGAGAC |
| 2 | rs28730786’ | *AKT1* | AGCCTGAGAGGAGCGCGTGA | CCAACCCTCCTTCACAATAG | CACGTCGCTCATGGTGCCC |
| 2 | rs41307094 | *AKT1* | AGAGTGAGGGGACACATGG | CCGATTCACGTAGGGAAATG | CATGTTAAGGACTTCTGCAGCTATG |
| 2 | rs4375597’ | *AKT1* | TATCAGGCTGCCCTGCTACT | CAGGACCTGGTTCCTGTAAG | ATTCCCTGGCTCTTCCAGCCTCA |
| 2 | rs55707948 | *AKT1* | TCCCCCTCAGATGATCTCTC | GCGACAGCGGAAAGGTTAAG | CGTCGAAAAGGTCAAGT |
| 2 | rs55874341’ | *AKT1* | CACATCATCTCGTACATGAC | TGCAGGTGCTGGAGGACAA | GGCAGGTGCTGGAGGACAATGACTA |
| 2 | rs58565216 | *AKT1* | CCTCCCTGACTCCCTGTGG | GTAAAAAAACGCCGTGGTGC | CTGGCCAGGAGGCGTGGA |
| 3 | rs11555429 | *AKT1* | CCTCCTGGCCAGACGCTG | GGGAAGGTTCCATATTATAT | CGTTGAATGTTGTAAAAAAACGCC |
| 3 | rs11555431 | *AKT1* | TGTAGCCTGTAGCTGGGATG | GGCCAAGTCCTTGCTTTCAG | GGCTGCTCAAGAAGGAC |
| 3 | rs17846826 | *AKT1* | TTCTCGGGTGCATTTGAGAG | GCTGCCCACAGCACAAAAAC | AAACGTCTTTCCATCTGGGCTC |
| 3 | rs2230506 | *AKT1* | CTGGAAAGAGTACTTCAGGG | CCACACACTCACCGAGAAC | CACTCACCGAGAACCGCGTC |
| 3 | rs2494735 | *AKT1* | CTTCCATGTGGAGACTCCTG | ATCCCCGTGTCCCTCCTAAG | AAGCGCTGGGGCTGCCCAAG |
| 3 | rs2494738 | *AKT1* | AGGACAGATGTGCCTGGGAT | TTTCTGTCGCTGGCCCTAAG | GCCCTAAGAAACAGCTCC |
| 3 | rs2498793 | *AKT1* | ACTGGCTGGTTTAGGGCTG | TGGAGTACCATGGACAGGAG | CCAGAGGTCTGGGCCAGTCTGC |
| 3 | rs2498796 | *AKT1* | AGCTCCCCTTGCATACCAC | AGAAACTGAGGCTTGGAGAG | GGAAGAGATGGGGCTTCC |
| 3 | rs34865723 | *AKT1* | TGCCACCAGGTTGAACTGAG | ATGTGTCCCCTCACTCTGTC | CTCTGTCAGCCAGCCGC |
| 3 | rs3803305 | *AKT1* | ATGGCCACCCCCACAGGGA | CGGTAGCACTTGACCTTTTC | AGTTCCGCTGTCGCCCCAGGCCCT |
| 3 | rs56217512 | *AKT1* | TGAGAGGAGCGCGTGAGCGT | GGTACTAACCTCGTTTGTGC | TCCAACCCTCCTTCACAATAGCCAC |
| 3 | rs56289559 | *AKT1* | TGGTCCTGGTTGTAGAAGGG | TGGAGGACAATGACTACGGC | TGGTCATGTACGAGATGATGTG |
| 4 | rs11555436 | *AKT1* | ATGATGGCACCTTCATTGGC | CACAGAGAAGTTGTTGAGGG | ACGTTGGTCCACATCCT |
| 4 | rs17102385 | *AKT1* | GAATGTTGTAAAAAAACGCC | TCCACGCCTCCTGGCCAGA | GGAATCCTGGCCAGACGCTGC |
| 4 | rs1804268 | *AKT1* | CTCTGTCCCACTGGGTAAAC | TAAGGCCGTGTCTCTGAGGA | TCTGAGGACGTCATCGGAG |
| 4 | rs2494732 | *AKT1* | TTTCAGGGCTGCTCAAGAAG | GATGAGGGGATGGAGGTGT | GATGGAGGTGTAGCCTG |
| 4 | rs2494749’ | *AKT1* | ACAGGCACTCACAGACCCT | ACAAACGAGGTTAGTACCCG | TTTGCCTGGGGAGGGAGAGA |
| 4 | rs34409589 | *AKT1* | TTCTTGAGGAGGAAGTAGCG | TAGAGTGTGCGTGGCTCTCA | CCCGCACGTCTGTAGGG |
| 4 | rs61761249 | *AKT1* | TCCCTACGTGAATCGGATTG | GGAAGAAAACTATCCTGCGG | CTCCGGGTGTGGCCTCAGCC |
| 5 | rs1154355 | *GRM7* | ATTGGAAATGGGATAATGGG | CTGGACAACACAGCAAGACC | GTACCACAGCAAGACCTTGTTTCCC |
| 5 | rs12630300 | *GRM7* | GGGCCACTTTTCAATTTGAG | GAAAGGTAAGGTAGTGCAAC | TGTAGTGCAACTCTTCCATA |
| 5 | rs17046224 | *GRM7* | GCTTAAGCGTATCTATTTAC | TAAGGGTTTGTGTGCTCAAG | AGGGGTTGTGTGCTCAAGTTTCTA |
| 5 | rs17046239 | *GRM7* | CACATGATCTTTGGGACAAC | TAAAGACTTCTGGGCTCCTG | GGCCCCTGAGGCTTTTCATATAAAC |
| 5 | rs191443 | *GRM7* | GAAGAAAGAGGCTCTCTAGG | ATAGCCTCCTACAAAGGGTG | CTCCTACAAAGGGTGCAATTA |
| 5 | rs340657 | *GRM7* | ATAGGTGTCACTGCCATTTG | TCCCACTCACCATCTGTTAG | TGTGGAGGCAGAGATTGTTATA |
| 5 | rs3846161 | *GRM7* | ATGGCCATCACATGAGAACG | ACAGAGCTGGATGGAGAAAG | GGATGGAGAAAGAATTCG |
| 5 | rs6443074 | *GRM7* | CCCTTGCTACATTCTGACTC | ATCCACCTGAATCAGATGCC | CATTCAATTCCAAGTCTATAGTG |
| 5 | rs7644436 | *GRM7* | CCACAATGAATGACCTTGAC | GGCAGATGTGTTTTTCCCTC | AATGAGGTTCAGAGAGGCTA |
| 5 | rs7651971 | *GRM7* | GGTAGGTGTTTAGCCCTACT | CATTTGCAGAGCCCTTGTTG | CCCTCAGAGCATTTAGT |
| 5 | rs983534 | *GRM7* | GTTAAGAGGTGGGTTCTTGC | AGGCTGAGGCAAGAGAATTG | AGGCCAGAAATTCGAGA |
| 5 | rs1124376 | *KAT2B* | ATCTTGGACAGCACAGTTGG | AGGAATGAAGAGAGGGAGAC | AGAGGGAGACAACATGGGGT |
| 5 | rs1610186 | *KAT2B* | GGGAAGTCTCAGTCATTATG | ACTTCCTCCACTGAGTCTTG | GGGATCCCAGTACAAAGTA |
| 5 | rs2929401 | *KAT2B* | CACCTGGCACATTAAATAAC | ACAAGCCTCAGATTTCGCAG | GGATCCTCAGATTTCGCAGCTGTAAT |
| 5 | rs11128927 | *RAB5A* | TGCGAGTGGCTCAGATCCGA | ATAACAATGGCGCTGGAGAG | GGGGAAGGGGAGAAAGGC |
| 5 | rs4241540 | *RAB5A* | AGCAGGAGACACCCACTTAG | CACTGATGTCCTTTGTATGC | AAAGGGTGGACCACTTGGTTGTCAGT |
| 5 | rs6790199 | *RAB5A* | TGAGGACCACTGTTCTAAGC | GATGATGGAACTGTACATGA | CCCGTACATGAAAATAACATTCCTCTT |
| 5 | rs6796538 | *VHL* | TCACTCTCGGCACCTCCTTG | AGAAAGGGGCTCCCACAGT | GGGTTGCAGCCGCATGCTGGAC |
| 6 | rs339807 | *GRM7* | GGATATGCCCCTCTTACTTG | AGCCAAGACTTGATCACAGG | GGGTGGATCAGTTCTCCTGAAACCAT |
| 6 | rs393046 | *GRM7* | TTTCAGTCTGGAGTGCAAGG | CTGTACATGTATCCCAGCAC | TACAACGATCTTCTCCC |
| 6 | rs4108607 | *GRM7* | CATAAATCCCTTTTCTGCCAC | GTACTGCAATAATGAAAGC | ACTGCAATAATGAAAGCAAATCC |
| 6 | rs9870018 | *GRM7* | ACCACACACCCAGAAATATG | TGGTCTTCATAATACCTCCC | GCTTTTCCTACATTTGCAC |
| 6 | rs9872244 | *GRM7* | GGGAAAAGCAGAGAGAAATG | TGAAACCTCTTAGGTTTGGG | GGGAAATTTCAATAGCAGA |
| 6 | rs9882058 | *GRM7* | GATCAATCTGATTAGGAGGAC | TGTTGGATACCAAAGACCAC | AACAATTTTAGTCCTTCAGAATCTA |
| 6 | rs12639078 | *KAT2B* | GGTAAAGGGTGGATGAACAG | AAAGAATTGCGCACTGTGCC | GCGAACTGTGCCCATTACCTAGACTCT |
| 6 | rs1915919 | *KAT2B* | ATTCTGTACATAGCTCGCCC | AGGAGTTGATCATGAAGGGC | AGACGAATGAGAACAGGACAC |
| 6 | rs2623074 | *KAT2B* | TTTTCAAGGTGCTTGAAAC | GCTTTCTTCTGAATTCAGGC | CCCCTTTCCAAAGCAGCACAT |
| 6 | rs2929402 | *KAT2B* | TAATCAGTCCCCATGCGTTG | TTCTGGAACCTCCTGCATAC | TTCCTACCACACAAACCTCGCAAAA |
| 6 | rs2929408 | *KAT2B* | GAGTGGGTAGCAAGAAATGG | ACAGGATTTGGCAAAGTGAC | GGAGTTGGCAAAGTGACAGCCCAG |
| 6 | rs13085694 | *RAB5A* | AGTAACCATCAGCTGCCTTC | GTCCTTCCTTTGCCCAAAGC | TCCTTCTCAAAAATGCAACTCTTATCT |
| 6 | rs2929344 | *RAB5A* | GGGTCAACATCTCTGGTATC | GCCAGTGTAAAGGTGATTGC | GGTGATTGCAAGATTCCA |
| 6 | rs2929346 | *RAB5A* | TCTTCCACCTCTCTCACTAC | ACAGTCCCTTGACTCAACTC | TATGCTTTGACCCAAAAAGA |
| 6 | rs7613136 | *RAB5A* | AAGGTTTCCATGAAGATGGG | AGCCTGGGCAACAGAGCAA | CAACAGAGCAAGACTCC |
| 6 | rs9810613 | *RAB5A* | TACATGGCTGGACATATGTG | CAAAGTAATTGTACTGTGA | AGTCATTGTACTGTGAACACCC |
| 6 | rs3773341 | *RAF1* | GCCAAACAGTTTGAAAACCC | AGTGGACGGAATAAAGTCTC | CCAGCAAACATTCTCCTAGCTCC |
| 7 | rs1154354 | *GRM7* | AATCCCATTATCCCATTTCC | TTGCTTGCATTGTGCCTGTG | CTGTACCCCCACAAACAAAAA |
| 7 | rs339805 | *GRM7* | TTTTTCCCCACTATTCAAGG | AGACTTCATTCCCAGTCTCC | TCCCAGTCTCCCCGACAAA |
| 7 | rs340660 | *GRM7* | CAGCCTGTCTCTGTCTGAAA | CCCCTGGAATATTTTTCAGC | TTCAGCAAACACAGTCC |
| 7 | rs3749380 | *GRM7* | GATCACTGTTGATCTGGTCC | CCTGCGGCGACATCAAGAG | CGGCGACATCAAGAGGGAAAA |
| 7 | rs9818072 | *GRM7* | AGGAAAGATAACCTTTGCCC | CCTTCTGAATATCTTTGCCC | GTATCTTTGCCCTCATCTTTATC |
| 7 | rs9876241 | *GRM7* | GAAGATGCTATGAAATTCAC | TAGCTAGAAGTGGGATTTGC | GTGGGATTTGCTTTCATTATTGC |
| 7 | rs9882865 | *GRM7* | ACATGTTTATGGGCTGGCTC | CCAAAACAGGCTTGGCTTAG | TTGGCTTAGAAGACACA |
| 7 | rs2948083 | *KAT2B* | CCCTTCTGCAGAAGGATTTC | GCCTCGCAGAGAATGAAAAC | CCTGGGGAATTCCTCTAGATAC |
| 7 | rs2948089 | *KAT2B* | TAAGGCACTCCCTTTACTCC | TAGCATGAGGCAACAGTCAC | AGGAGGCAACAGTCACCATATCTTA |
| 7 | rs6806287 | *KAT2B* | TCGTACCCAATCAAGTCCAG | GCAGGAATCTTAACCTGGAG | TCTTAACCTGGAGAGTGACA |
| 7 | rs4858660 | *RAB5A* | GCCCAGGTTATTTAACCCAG | ACTGCTAGATCATGTCCACC | CTTTAAGCATTTCCCAAAGTATAT |
| 7 | rs713178 | *RAF1* | TAGACATGGCTCAGAGTCGG | GGTTAAACTGCAGTGGAGTC | GGATTCAGTGGAGTCAGAGTCCGT |
| 7 | rs9855183 | *RAF1* | GGATGGGGTAAGTGCTTTTG | ACACTCCTGGGAAGGAGAAC | GGCAGGCAGCAGGGGCAC |
| 7 | rs265318 | *VHL* | TAGGGATGTAGGCATTTCTG | AGATTCATGCAAACATCCCC | CATCCATGCAAACATCCCCTCAGTT |
| 8 | rs1240966 | *GRM7* | ATTTGCTTGCATTGTGCCTG | AATCCCATTATCCCATTTCC | CTTGAGATATTTTTGTTTGTGGG |
| 8 | rs2948097 | *KAT2B* | CTAAGGGTCCAGCTTCATTC | GCCAAGACCATTCAATAGAG | GCTGGAAAAACTAGGTATCTG |
| 8 | rs13072891 | *RAB5A* | ATGTTGGCTGCCTATTATTC | CAAGAGCAAAAATCTGCCTC | TCCTGTGGAGAAATATGTT |
| 9 | rs12487836 | *GRM7* | CTAAATGTGAGTGCCAGATTC | TTGCAGTGTGCCATCCATTG | GTCCATTTGACAGATCTATTCAGTA |
| 9 | rs17046322 | *GRM7* | TATTTTTTTCCTGGGCCCTG | GGGCTAATGCCCACATCAAG | GCCCACATCAAGTTAATTG |
| 9 | rs340655 | *GRM7* | TTTGGTGAGTTTGTGGGAAC | CAAAAGCAGTCTGAATACAGG | TTGACTAGTAATGACCAAGA |
| 9 | rs414907’ | *GRM7* | CTCTTAGCCTTGCTGTTATC | TTAGTGACATTACTCAGTGG | CCTCAGTGGAATGAAACTTTTT |
| 9 | rs421802 | *GRM7* | CCACAAATGTAAATACAAAG | GCTTCAGAAGTGTGAAATTAC | TGCAGAAGTGTGAAATTACAAACATG |
| 9 | rs7651591 | *GRM7* | GGTGGACTTCCACAAGTTTC | GCCACAATGATCGATTAGTC | CTGTGTGTGTGTGTGTATTAG |
| 9 | rs2929404 | *KAT2B* | TAGCTAGCTTTGTGGTCTGC | AAGTCTGCCATGAACCAAGG | CGGGAAACACACTTAACTATGAGTA |
| 9 | rs6765791 | *KAT2B* | AAGGACATAGCACTCACTGG | TGGCATAAGAAGTCACCCAC | GTCAGGTGGAAAGAGCAA |
| 9 | rs9874923 | *KAT2B* | GCCCATCAACTAGTTAACGC | GTTGTAGCATATGTCAGCAC | CATATGTCAGCACTTCTTTAT |
| 9 | rs2127956 | *RAB5A* | CGTGAGTGTAGTTAGGGAAG | TCCTGGGTGCTGAGTAATAC | GGTTATTACTCTTCCTCACATTAATTG |
| 9 | rs6778866 | *RAB5A* | AAGCCTAGAGATGCAGTACC | GTTAGCCCAGAGATTAGCAG | GGAGATTAGCAGGGAGCAG |
| 9 | rs9858341 | *RAB5A* | GTGTTTTAAATTGAAAAAGC | GCAGAGTCATGAGTTGAACTT | TCGCCAACATATATTCCATCTAG |
| 9 | rs2442809’ | *RAF1* | CGACAGAACGAGACTCTATC | TGCTAGGGATTACAGGTGTG | CCACCACACCTGGCCTA |
| 9 | rs2596831 | *RAF1* | GATGGCCCAGTGAACTAATG | CCCCAGTCCCAAAAACAAAC | GTGGCATTCTGAGCAAA |
| 9 | rs5746223 | *RAF1* | AACCCATGTCATCTGTTTCC | GCCAGTGCTATAACTAAAATG | AGACAAAAATTGGAAGTATGGTTA |
| 9 | rs7956 | *RAF1* | GCTGATGGCAAGTCTTGTAG | TTGGAACAAGCTAGTGCTGG | AGCTAGTGCTGGTTCACAAGGTT |
| 9 | rs388600 | *VHL* | GTCAGTGGTTTGCAACCCTG | GTAGGGCTAGGCATTTATAC | TTAAAAGCACCCCAGATGTTTCTA |
| 10 | rs163540 | *GRM7* | GGCTGCAAGTAACAGAAAAC | CCCCAATGCTTCCTCTTTTG | CTGCTCATTGCATAGGT |
| 10 | rs9838115 | *GRM7* | ACACTGTGGAATCAGCTAGG | GAAGTCTGGTACTGGTTTGC | GGTTTTGCCTGCTATACGGAGGGG |
| 10 | rs4858754 | *KAT2B* | ACTCTCTGAACACTAGCTGG | TAACCTGTGGGATGTGATGC | TTGGGATGTGATGCTATCTCCA |
| 10 | rs10510497 | *RAB5A* | TTTCCTGCTTCACCCATCAC | GAATGAGAGAAGAGGAAAAG | AGAAACTTTATGTGGTGGTCAG |
| 10 | rs11128930 | *RAB5A* | CAGTGCACTCATAACAACTC | GATTAAGAGGATGAAGGAGG | ATGAAGGAGGTAAACTAGAA |
| 10 | rs12488378 | *RAB5A* | GCAATCCGATCTCAAAGCAG | GGACAGACCACTATGGTAAG | ATAGCTTTGACTCAGGTTACT |
| 10 | rs13081007 | *RAB5A* | GCTTCTGAAGGTTCTCTCTG | TCACCTTTACACTGGCCTTC | AGCCCACTGCAGTCTCTC |
| 10 | rs2454436 | *RAF1* | TTAACGGCAGGGATACCTTC | GGCTGTACCTTCTTGGTTTG | CTTTATGATGTGTGCATGA |
| 10 | rs3729931 | *RAF1* | AGGGAAAGAAAACAGCTGAG | TAAGCAGCTAGAGGGTTAGG | ACTTGTTATTATCTGTTGTTCATT |
| 10 | rs166538 | *VHL* | AAACCGTATCAGGGCTGTTC | CTCAAAATCCTCCCTACTGC | CCCGAGTTGCTCCACGCCAG |
| 11 | rs11555433 | *AKT1* | TTGAGTACCTGAAGCTGCTG | GATCTTCATGGCGTAGTAGC | TAGCGGCCTGTGGCCTTCTCCTTC |
| 11 | rs2494749 | *AKT1* | ACAGGCACTCACAGACCCT | ACAAACGAGGTTAGTACCCG | GGGCCTGGGGAGGGAGAGA |
| 11 | rs28730786 | *AKT1* | CCAACCCTCCTTCACAATAG | AGCCTGAGAGGAGCGCGTGA | GTGAGCGTCGCGGGAGCCT |
| 11 | rs4375597 | *AKT1* | TATCAGGCTGCCCTGCTACT | CAGGACCTGGTTCCTGTAAG | CCTGGCTCTTCCAGCCTCA |
| 11 | rs55874341 | *AKT1* | CACATCATCTCGTACATGAC | TGCAGGTGCTGGAGGACAA | CAGGTGCTGGAGGACAATGACTA |
| 11 | rs414907 | *GRM7* | CTCTTAGCCTTGCTGTTATC | TTAGTGACATTACTCAGTGG | TTACTCAGTGGAATGAAACTTTTT |
| 11 | rs11621560 | *HSP90AA1* | GTTGTTGAGGAAGGAAGAGG | AACCCCAAGTTCTTCCACTC | TTCCACTCCTGCTTCAC |
| 11 | rs1190583 | *HSP90AA1* | GCAAAACTACATCTCCTCAC | ACATCTTTTCACAATCGAGG | TTCACAATCGAGGAAGTTTAGA |
| 11 | rs2298877 | *HSP90AA1* | GCTGCTTGGAGGTATTAAAG | CTTAAAGCCAGCTATTAGGG | CCAGCTATTAGGGTAATACTC |
| 11 | rs7160651 | *HSP90AA1* | GGAAGTCTGCACTGAAACTC | CTATGTGGCCAATTGACCAG | TCAGGCCACCTTGTCAC |
| 11 | rs8005080 | *HSP90AA1* | CAGGAATACCCCAACCAAGG | TATCTCCTTCCCAGAGCTTC | CCTTCCCAGAGCTTCTCCGAAC |
| 11 | rs8005905 | *HSP90AA1* | GTACCAAGAAAAGGCCCAAG | TCCAGAGACAGAGTAGAGTG | GGTGGATCCAGACACCA |
| 12 | rs17846829 | *AKT1* | TTAACCTTTCCGCTGTCGCC | GCAGCGGCAGCGTCTGGC | AGCGTCTGGCCAGGAGG |
| 12 | rs2498791 | *AKT1* | CTGAGCCTGCTCTGCTCTC | TCCCCTCCCCCAGCCACAG | GCAGCGGGCAGGCAGGGGC |
| 12 | rs1190584 | *HSP90AA1* | TCCTCGATTGTGAAAAGATG | TTATATACTCCGAGCACTT | CTCCGAGCACTTCAGAAT |
| 12 | rs2442809 | *RAF1* | CGACAGAACGAGACTCTATC | TGCTAGGGATTACAGGTGTG | CCACCACACCTGGCCTA |
| R | rs11555433 | *AKT1* | GATCTTCATGGCGTAGTAGC | TTGAGTACCTGAAGCTGCTG | TAGCGGCCTGTGGCCTTCTCCTTC |
| R | rs28730786 | *AKT1* | AGCCTGAGAGGAGCGCGTGA | CCAACCCTCCTTCACAATAG | GTGAGCGTCGCGGGAGCCT |
| R | rs4375597 | *AKT1* | CAGGACCTGGTTCCTGTAAG | TATCAGGCTGCCCTGCTACT | CCTGGCTCTTCCAGCCTCA |
| R | rs55874341 | *AKT1* | TGCAGGTGCTGGAGGACAA | CACATCATCTCGTACATGAC | CAGGTGCTGGAGGACAATGACTA |
| R | rs2494749 | *AKT1* | ACAAACGAGGTTAGTACCCG | ACAGGCACTCACAGACCCT | GGGCCTGGGGAGGGAGAGA |
| R | rs414907 | *GRM7* | TTAGTGACATTACTCAGTGG | CTCTTAGCCTTGCTGTTATC | TTACTCAGTGGAATGAAACTTTTT |
| R2 | rs2442809 | *RAF1* | TGCTAGGGATTACAGGTGTG | CGACAGAACGAGACTCTATC | CCACCACACCTGGCCTA |

‘Indicates that this SNP failed quality control filters and was re-typed in primer set R or R2.
