# Supplementary material for: Genomewide Analysis of Inherited Variation Associated with Phosphorylation of PI3K/AKT/mTOR Signaling Proteins
Source: PLoS One. 2011 Sep 19;6(9):e24873. doi: 10.1371/journal.pone.0024873 (PMC3176272; doi:10.1371/journal.pone.0024873)
Supplement: Table S2 — All association test results for SNPs on chromosomes 14 and 3. (DOC) [file pone.0024873.s007.doc]

**Table S2.** **All association test results for SNPs on chromosomes 14 and 3**

| Phenotype | SNP | Gene | Additive | Dominant | Recessive |
| --- | --- | --- | --- | --- | --- |
| AKT1 ratio | rs10138227 | *AKT1* | 0.266 | 0.192 | 0.789 |
| AKT1 ratio | rs1130214 | *AKT1* | 0.147 | 0.135 | 0.696 |
| AKT1 ratio | rs1130233 | *AKT1* | 0.845 | 0.125 | 0.421 |
| AKT1 ratio | rs11848899 | *AKT1* | 0.777 | 0.777 | - |
| AKT1 ratio | rs12588965 | *AKT1* | 0.042 | - | 0.042 |
| AKT1 ratio | rs2494732 | *AKT1* | 0.204 | 0.106 | 0.635 |
| AKT1 ratio | rs2494738 | *AKT1* | 0.423 | 0.200 | 0.149 |
| AKT1 ratio | rs2494746 | *AKT1* | 0.832 | 0.586 | 0.997 |
| AKT1 ratio | rs2494748 | *AKT1* | 0.193 | 0.951 | 0.163 |
| AKT1 ratio | rs2494749 | *AKT1* | 0.954 | 0.155 | 0.543 |
| AKT1 ratio | rs2498796 | *AKT1* | 0.659 | 0.785 | 0.069 |
| AKT1 ratio | rs3803304 | *AKT1* | 0.425 | 0.310 | 0.552 |
| AKT1 ratio | rs41307094 | *AKT1* | 0.251 | - | 0.251 |
| AKT1 ratio | rs11621560 | *HSP90AA1* | 0.898 | 0.867 | 0.963 |
| AKT1 ratio | rs1190583 | *HSP90AA1* | 0.211 | 0.211 | - |
| AKT1 ratio | rs1190584 | *HSP90AA1* | 0.0042* | 0.564 | 0.0036* |
| AKT1 ratio | rs2298877 | *HSP90AA1* | 0.216 | 0.826 | 0.171 |
| AKT1 ratio | rs7160651 | *HSP90AA1* | 0.518 | - | 0.518 |
| AKT1 ratio | rs8005080 | *HSP90AA1* | 0.557 | - | 0.557 |
| AKT1 ratio | rs8005905 | *HSP90AA1* | 0.136 | 0.136 | - |
| p70S6K ratio | rs1154354 | *GRM7* | 0.831 | 0.465 | 0.872 |
| p70S6K ratio | rs1154355 | *GRM7* | 0.922 | 0.688 | 0.408 |
| p70S6K ratio | rs1240966 | *GRM7* | 0.506 | 0.506 | - |
| p70S6K ratio | rs12487836 | *GRM7* | 0.408 | 0.585 | 0.466 |
| p70S6K ratio | rs12630300 | *GRM7* | 4.10 × 10^-4^ * | - | 4.10 × 10^-4^ * |
| p70S6K ratio | rs163540 | *GRM7* | 0.574 | 0.574 | - |
| p70S6K ratio | rs17046224 | *GRM7* | 0.681 | - | 0.681 |
| p70S6K ratio | rs17046239 | *GRM7* | - | - | - |
| p70S6K ratio | rs17046322 | *GRM7* | - | - | - |
| p70S6K ratio | rs191443 | *GRM7* | 0.022 | 0.046 | 0.076 |
| p70S6K ratio | rs339805 | *GRM7* | 0.025 | 0.211 | 0.040 |
| p70S6K ratio | rs339807 | *GRM7* | 0.538 | 0.382 | 0.442 |
| p70S6K ratio | rs340655 | *GRM7* | 0.003 | 0.124 | 0.012 |
| p70S6K ratio | rs340657 | *GRM7* | 0.010 | 0.037 | 0.105 |
| p70S6K ratio | rs340660 | *GRM7* | 0.152 | 0.555 | 0.030 |
| p70S6K ratio | rs3749380 | *GRM7* | 0.134 | 0.598 | 0.004 |
| p70S6K ratio | rs3846161 | *GRM7* | 0.650 | 0.970 | 0.307 |
| p70S6K ratio | rs393046 | *GRM7* | 0.005 | 0.113 | 0.005 |
| p70S6K ratio | rs4108607 | *GRM7* | 0.085 | 0.150 | 0.441 |
| p70S6K ratio | rs421802 | *GRM7* | 0.053 | 0.089 | 0.211 |
| p70S6K ratio | rs6443074 | *GRM7* | 0.944 | - | 0.944 |
| p70S6K ratio | rs7644436 | *GRM7* | 0.060 | 0.408 | 0.059 |
| p70S6K ratio | rs7651591 | *GRM7* | 0.876 | 0.078 | 0.660 |
| p70S6K ratio | rs7651971 | *GRM7* | 0.131 | 0.017 | 0.776 |
| p70S6K ratio | rs9818072 | *GRM7* | 0.796 | - | 0.796 |
| p70S6K ratio | rs9838115 | *GRM7* | 0.401 | - | 0.401 |
| p70S6K ratio | rs9870018 | *GRM7* | 0.629 | 0.957 | 0.486 |
| p70S6K ratio | rs9872244 | *GRM7* | 0.877 | 0.645 | 0.980 |
| p70S6K ratio | rs9876241 | *GRM7* | 0.151 | 0.822 | 0.145 |
| p70S6K ratio | rs9882058 | *GRM7* | 0.127 | 0.445 | 0.156 |
| p70S6K ratio | rs9882865 | *GRM7* | 0.074 | 0.093 | 0.401 |
| p70S6K ratio | rs1124376 | *KAT2B* | 0.785 | - | 0.785 |
| p70S6K ratio | rs12639078 | *KAT2B* | 0.135 | 0.019 | 0.508 |
| p70S6K ratio | rs1610186 | *KAT2B* | 0.584 | 0.358 | 0.902 |
| p70S6K ratio | rs1915919 | *KAT2B* | 0.297 | 0.172 | 0.622 |
| p70S6K ratio | rs2623074 | *KAT2B* | 0.140 | 0.221 | 0.216 |
| p70S6K ratio | rs2929401 | *KAT2B* | 0.974 | 0.323 | 0.284 |
| p70S6K ratio | rs2929402 | *KAT2B* | 0.471 | 0.475 | 0.674 |
| p70S6K ratio | rs2929404 | *KAT2B* | 0.538 | 0.199 | 0.207 |
| p70S6K ratio | rs2929408 | *KAT2B* | 0.208 | 0.320 | 0.216 |
| p70S6K ratio | rs2948083 | *KAT2B* | 0.903 | 0.896 | 0.528 |
| p70S6K ratio | rs2948089 | *KAT2B* | 0.780 | - | 0.780 |
| p70S6K ratio | rs2948097 | *KAT2B* | 0.477 | 0.229 | 0.633 |
| p70S6K ratio | rs4858754 | *KAT2B* | 0.570 | 0.728 | 0.183 |
| p70S6K ratio | rs6765791 | *KAT2B* | 0.289 | 0.449 | 0.294 |
| p70S6K ratio | rs6806287 | *KAT2B* | 0.488 | - | 0.488 |
| p70S6K ratio | rs9874923 | *KAT2B* | 0.963 | 0.966 | 0.728 |
| p70S6K ratio | rs10510497 | *RAB5A* | 1.000 | 0.837 | 0.501 |
| p70S6K ratio | rs11128927 | *RAB5A* | 0.992 | 0.388 | 0.584 |
| p70S6K ratio | rs11128930 | *RAB5A* | 0.826 | 0.426 | 0.492 |
| p70S6K ratio | rs12488378 | *RAB5A* | 0.862 | 0.952 | 0.769 |
| p70S6K ratio | rs13072891 | *RAB5A* | 0.960 | 0.501 | 0.546 |
| p70S6K ratio | rs13081007 | *RAB5A* | 0.184 | 0.345 | 0.138 |
| p70S6K ratio | rs13085694 | *RAB5A* | 0.784 | 0.428 | 0.499 |
| p70S6K ratio | rs2127956 | *RAB5A* | 0.913 | 0.604 | 0.330 |
| p70S6K ratio | rs2929344 | *RAB5A* | 0.813 | 0.291 | 0.422 |
| p70S6K ratio | rs2929346 | *RAB5A* | 0.814 | 0.240 | 0.122 |
| p70S6K ratio | rs4241540 | *RAB5A* | 0.838 | 0.229 | 0.729 |
| p70S6K ratio | rs4858660 | *RAB5A* | 0.915 | 0.807 | 0.930 |
| p70S6K ratio | rs6778866 | *RAB5A* | 0.779 | - | 0.779 |
| p70S6K ratio | rs6790199 | *RAB5A* | 0.560 | 0.560 | - |
| p70S6K ratio | rs7613136 | *RAB5A* | 0.849 | 0.774 | 0.975 |
| p70S6K ratio | rs9810613 | *RAB5A* | 0.848 | 0.502 | 0.994 |
| p70S6K ratio | rs9858341 | *RAB5A* | 0.922 | 0.525 | 0.102 |
| p70S6K ratio | rs2454436 | *RAF1* | 0.463 | 0.621 | 0.529 |
| p70S6K ratio | rs2596831 | *RAF1* | 0.163 | 0.319 | 0.224 |
| p70S6K ratio | rs3729931 | *RAF1* | 0.391 | 0.832 | 0.113 |
| p70S6K ratio | rs3773341 | *RAF1* | 0.178 | 0.699 | 0.048 |
| p70S6K ratio | rs5746223 | *RAF1* | 6.35 × 10^-5^ ** | 6.35 × 10^-5^ ** | - |
| p70S6K ratio | rs713178 | *RAF1* | 4.57 × 10^-4^ * | 5.65 × 10^-4^ * | 0.356 |
| p70S6K ratio | rs7956 | *RAF1* | 0.384 | 0.106 | 0.199 |
| p70S6K ratio | rs9855183 | *RAF1* | 6.35 × 10^-5^ ** | 6.35 × 10^-5^ ** | - |
| p70S6K ratio | rs166538 | *VHL* | 0.449 | 0.859 | 0.345 |
| p70S6K ratio | rs265318 | *VHL* | 0.067 | 0.077 | 0.534 |
| p70S6K ratio | rs388600 | *VHL* | 0.121 | 0.427 | 0.120 |

* Significant using false discovery rate of 5%

** Significant at Bonferroni-corrected threshold
